# Motor imagery-based brain–computer interface rehabilitation programs enhance upper extremity performance and cortical activation in stroke patients

**DOI:** 10.1186/s12984-024-01387-w

**Published:** 2024-05-29

**Authors:** Zhen-Zhen Ma, Jia-Jia Wu, Zhi Cao, Xu-Yun Hua, Mou-Xiong Zheng, Xiang-Xin Xing, Jie Ma, Jian-Guang Xu

**Affiliations:** 1 Department of Rehabilitation Medicine, Shuguang Hospital, Shanghai University of Traditional Chinese Medicine, Shanghai, China; 2 Department of Rehabilitation Medicine, Yueyang Hospital of Integrated Traditional Chinese and Western Medicine, Shanghai University of Traditional Chinese Medicine, Shanghai, China; 3 Department of Tuina, Yueyang Hospital of Integrated Traditional Chinese and Western Medicine, Shanghai University of Traditional Chinese Medicine, Shanghai, China; 4 Department of Trauma and Orthopedics, Yueyang Hospital of Integrated Traditional Chinese and Western Medicine, Shanghai University of Traditional Chinese Medicine, Shanghai, China; 5 School of Rehabilitation Science, Shanghai University of Traditional Chinese Medicine, Shanghai, China; 6 Engineering Research Center of Traditional Chinese Medicine Intelligent Rehabilitation, Ministry of Education, Shanghai, China; 7 Rehabilitation Center, Qilu Hospital of Shandong University, Jinan, China

**Keywords:** Brain–computer interface (BCI), Fugl–Meyer Assessment of the Upper Extremity (FMA-UE), Motor imagery (MI), Stroke rehabilitation, fMRI

## Abstract

**Background:**

The most challenging aspect of rehabilitation is the repurposing of residual functional plasticity in stroke patients. To achieve this, numerous plasticity-based clinical rehabilitation programs have been developed. This study aimed to investigate the effects of motor imagery (MI)-based brain–computer interface (BCI) rehabilitation programs on upper extremity hand function in patients with chronic hemiplegia.

**Design:**

A 2010 Consolidated Standards for Test Reports (CONSORT)-compliant randomized controlled trial.

**Methods:**

Forty-six eligible stroke patients with upper limb motor dysfunction participated in the study, six of whom dropped out. The patients were randomly divided into a BCI group and a control group. The BCI group received BCI therapy and conventional rehabilitation therapy, while the control group received conventional rehabilitation only. The Fugl–Meyer Assessment of the Upper Extremity (FMA-UE) score was used as the primary outcome to evaluate upper extremity motor function. Additionally, functional magnetic resonance imaging (fMRI) scans were performed on all patients before and after treatment, in both the resting and task states. We measured the amplitude of low-frequency fluctuation (ALFF), regional homogeneity (ReHo), z conversion of ALFF (zALFF), and z conversion of ReHo (ReHo) in the resting state. The task state was divided into four tasks: left-hand grasping, right-hand grasping, imagining left-hand grasping, and imagining right-hand grasping. Finally, meaningful differences were assessed using correlation analysis of the clinical assessments and functional measures.

**Results:**

A total of 40 patients completed the study, 20 in the BCI group and 20 in the control group. Task-related blood-oxygen-level-dependent (BOLD) analysis showed that when performing the motor grasping task with the affected hand, the BCI group exhibited significant activation in the ipsilateral middle cingulate gyrus, precuneus, inferior parietal gyrus, postcentral gyrus, middle frontal gyrus, superior temporal gyrus, and contralateral middle cingulate gyrus. When imagining a grasping task with the affected hand, the BCI group exhibited greater activation in the ipsilateral superior frontal gyrus (medial) and middle frontal gyrus after treatment. However, the activation of the contralateral superior frontal gyrus decreased in the BCI group relative to the control group. Resting-state fMRI revealed increased zALFF in multiple cerebral regions, including the contralateral precentral gyrus and calcarine and the ipsilateral middle occipital gyrus and cuneus, and decreased zALFF in the ipsilateral superior temporal gyrus in the BCI group relative to the control group. Increased zReHo in the ipsilateral cuneus and contralateral calcarine and decreased zReHo in the contralateral middle temporal gyrus, temporal pole, and superior temporal gyrus were observed post-intervention. According to the subsequent correlation analysis, the increase in the FMA-UE score showed a positive correlation with the mean zALFF of the contralateral precentral gyrus (r = 0.425, P < 0.05), the mean zReHo of the right cuneus (r = 0.399, P < 0.05).

**Conclusion:**

In conclusion, BCI therapy is effective and safe for arm rehabilitation after severe poststroke hemiparesis. The correlation of the zALFF of the contralateral precentral gyrus and the zReHo of the ipsilateral cuneus with motor improvements suggested that these values can be used as prognostic measures for BCI-based stroke rehabilitation. We found that motor function was related to visual and spatial processing, suggesting potential avenues for refining treatment strategies for stroke patients.

*Trial registration*: The trial is registered in the Chinese Clinical Trial Registry (number ChiCTR2000034848, registered July 21, 2020).

## Introduction

Brain–computer interface (BCI) systems act as relay stations that transmit brain signals to output devices to perform desired actions [1, 2]. Noninvasive BCI systems may allow individuals to replace or restore lost or impaired function and have been widely used in the functional recovery of people with neuromuscular diseases or injuries, including amyotrophic lateral sclerosis (ALS), spinal cord injury, stroke, or cerebral palsy [3]. Electroencephalography (EEG) is widely used in the development of noninvasive BCI systems due to its noninvasiveness, ease of use, and low cost. EEG-based BCI signal types include stimulus-evoked potentials, slow cortical potentials, and sensorimotor rhythms (SMRs). During motor attempts or motor imagery (MI), the amplitude of the SMR decreases, a modulation called event-related desynchronization (ERD), which can be translated into control commands for external devices. No actual physical movement is necessary for controlling BCI-based devices, and even stroke survivors with severe chronic motor deficits can modulate or manipulate them. The generated control commands during motor attempts or imagination are independent of residual motor function [4]. Therefore, MI tasks can modulate SMRs generated by neuronal units of the sensorimotor gyrus and have been a powerful paradigm for noninvasive BCI systems to achieve movement restoration in stroke survivors. An enormous amount of research has shown that a BCI system based on MI (MI-BCI) can improve motor outcomes in stroke survivors and may represent a promising rehabilitation approach for functional recovery after stroke [5–7].

BCI training based on MI is a closed-loop neural circuit intervention method and is a popular topic of interest in research on robot-assisted rehabilitation. Unlike traditional open-loop stimulation, closed-loop stimulation can titrate charge delivery to the brain through feedback from relevant biomarkers of neurological symptoms, thereby enhancing the amount of stimulation delivered and therapeutic efficacy [8]. Continuous (i.e., adaptive) or on–off (i.e., on-demand) methods can be used based on feedback from biomarkers associated with the patient's neurological symptoms (e.g., seizure events, tremor episodes, or mood changes), thereby reducing side effects and delivering the appropriate amount of stimulation, enhancing the therapeutic effect. EEG features during MI characterize the feedback of the patient's real-time neural activity similar to performing motor tasks or observing during actual tasks. The common features include the modulation of spectral features at the alpha (8 to 12 Hz) and beta (18–26 Hz) frequency bands, event-related desynchronization (ERD), and event-related synchronization (ERS). MI is not completely dependent on the patient’s residual motor function performance; it reshapes the patient's motor intention to improve motor output and is an active type of rehabilitation training [9].

MI-BCI technology, which combines visual, auditory, proprioceptive, and cognitive activities through the interaction of multisensory stimuli, increases external feedback input, and stimulates changes in neural remodeling patterns, has been shown to effectively improve motor function [10]. Recent controlled trials have shown the potential benefit of BCI-based therapies in the motor rehabilitation of stroke survivors. Ramos-Murguialday et al. [11] were the first team to conduct a randomized controlled study of BCI training in stroke patients, and the study verified that BCI systems can significantly improve the training effect of patients with severe upper limb paralysis. In addition, BCI technology combines virtual limbs and functional electrical stimulation (FES) as feedback to provide patients with closed-loop sensorimotor integration for motor rehabilitation, which has a significant effect on poststroke rehabilitation [12]. Similarly, Biasiucci et al. [13] recently presented the results of a randomized controlled trial in which 14 stroke patients who received BCI training combined with FES were compared with 13 patients who received sham FES. Compared with those in the sham group, chronic stroke survivors who received BCI training showed significant clinically relevant improvements and lasting motor recovery. An increase in functional connectivity between motor areas in the affected hemisphere is significantly correlated with functional improvement. Furthermore, the EEG signal is used as the characteristic recognition signal of the BCI in stroke patients, which can provide insight into brain remodeling patterns and help optimize clinical strategies for BCI training. A neurophysiologic EEG study by Li et al. [14] reported that MI-based BCI training simultaneously activated the bilateral cerebral hemispheres, and the event-related desynchronization (ERD) of the affected sensorimotor cortices (SMCs) was significantly enhanced, which enhanced the motor function of the upper extremity in stroke patients by inducing optimal cerebral motor functional reorganization. In addition, Guo et al. [15] recently proposed another noninvasive BCI paradigm, steady-state visually evoked potential (SSVEP)-based BCI. The detection of user intention can trigger soft robotic gloves for poststroke hand function rehabilitation. In this randomized controlled trial, 30 poststroke patients with impaired upper limb dysfunction were randomly and equally divided into three groups: control, robotic, and SSVEP-BCI groups. The recovery of hand function after rehabilitation with the SSVEP-BCI-controlled soft robotic glove was better than that achieved with robotic glove rehabilitation alone, and the efficacy was equivalent to that achieved with previously reported MI-BCI robotic hand rehabilitation methods.

Despite the promising results achieved thus far, BCI-based stroke rehabilitation is still a new field [13], and the mechanisms of BCI-based therapies remain largely unclear. It was hypothesized that continuity between suitable motor-related cortical activity and rich afferent feedback is the basic mechanism by which the BCI increases beneficial functional activity-dependent plasticity to achieve clinically important outcomes. In previous studies, more attention has been given to the clinical outcomes of BCI training and remodeling patterns on resting-state functional magnetic resonance imaging (fMRI), and little attention has been given to the exploration of patient biomarkers during the MI-BCI task. In this study, we evaluated the safety and efficacy of MI-BCI therapy in disabled chronic stroke patients, assessed the strength of clinically relevant functional recovery, and investigated the association between features of functional neuroplasticity and motor improvement. Neuroplasticity alterations were detected by fMRI, a neural detection technique that has been widely used to capture patterns of brain activity that may be localized or widely distributed. Therefore, we referred to our method as "region-based analysis", which included resting-state and task-state seeking to characterize discrete and clearly delineated brain regions. Our findings can provide physiological indicators of dynamic physiological information about brain function and contribute to new constructive directions for further design and improvement of MI-BCI.

## Materials and methods

### Study design

The research adhered to the Declaration of Helsinki (2008) and received ethics approval from the Medical Ethics Committee of Yueyang Hospital with Clinical Trial Registration number ChiCTR2000034848, registered on July 21, 2020. The sample size calculation was informed by past research studies that employed comparable endpoints [16]. A total of forty-six individuals who had suffered from strokes were evaluated for qualification between the months of March 2020 and January 2021. After registration, individuals were randomly assigned to the control or BCI group through a computer-generated random sequence. Six patients withdrew during the intervention or follow-up. The remaining 40 patients completed the training and MRI scans. All participants provided written consent after receiving detailed information about the research study. The eligibility requirements for participants align with the selection criteria used in our previous research project [17]. The subjects underwent motor function assessment via the Fugl–Meyer assessment (FMA) and corticospinal excitability assessment via fMRI pre- and postintervention. All screening and research procedures were conducted at Yueyang Hospital in Shanghai.

### Clinical assessments

The assessment of motor function in the affected upper limb using the Fugl-Meyer Assessment of the Upper Extremity (FMA-UE) was conducted on all stroke patients at the initial screening, pre-treatment, and post-treatment follow-up after 2 weeks. The FMA-UE measures motor recovery in different stages [18], focusing on reflexes, arm movement patterns, and hand dexterity. The motor assessment scores each item based on a 3-point ordinal scale to determine the total motor score for the affected side, ranging from 0 (hemiplegia) to 66 (normal) [19].

### MI-BCI and conventional interventions

The two groups participated in standard physical therapy and occupational therapy sessions for a fortnight, emphasizing exercises, muscle tension control, and limb control drills to enhance functionality, balance, and daily activities. In addition, each subject in the BCI cohort participated in ten 40-min MI-BCI training sessions distributed over 2 weeks, utilizing the MI-based BCI technology [17]. Every treatment session was conducted by a skilled and knowledgeable physical therapist. A single member of the occupational therapy team was kept unaware of the allocation and intervention during the assessments and data analysis process.

### MRI

The data from the MRI scans were obtained with a 3T scanner manufactured by Siemens AG (MAGNETOM Verio) with an 8-channel head array coil for two sessions: pretreatment and posttreatment. Each fMRI scan included a resting-state session and two task-state sessions with a block design. During the resting-state imaging, the scanner was used to measure brain activity. Employing the interleaved scanning order, the parameters selected for use were as follows: number of slices = 43, TR = 3000 ms, matrix size = 64 × 64, FA = 90°, Field of view = 192 × 192 mm^2^, voxel size = 3 × 3 × 3 mm^3^, and number of acquisitions = 200. For task-state imaging, the echo-planar imaging protocol (TR/TE = 3000/35 ms, FOV = 220 × 220 mm^2^, 39 axial slices, acquisition matrix = 64 × 64, voxel size = 3 × 3 × 3 mm^3^, number of acquisitions = 100) was used to obtain the fMRI data. The fMRI data were measured with an echo-planar imaging sequence (TR/TE = 3000/35 ms, FOV = 220 × 220 mm^2^, 39 axial slices, acquisition matrix = 64 × 64, voxel size = 3 × 3 × 3 mm^3^, number of acquisitions = 100). High-resolution whole-brain anatomical scans were conducted on all participants to serve as a reference for functional activation mapping. The scans were 3D T1-weighted with a repetition time of 1900 ms. The time required for each echo (TE) is 2.93 ms, the flip angle is set at 9°, the field of view is 240 mm × 240 mm, the acquisition matrix is 256 × 256, the acquisition is done sagittally, the spatial resolution is 1 × 1 × 1 mm^3^, and there is no interslice space.

### fMRI experimental paradigm

**Fig. 1:**
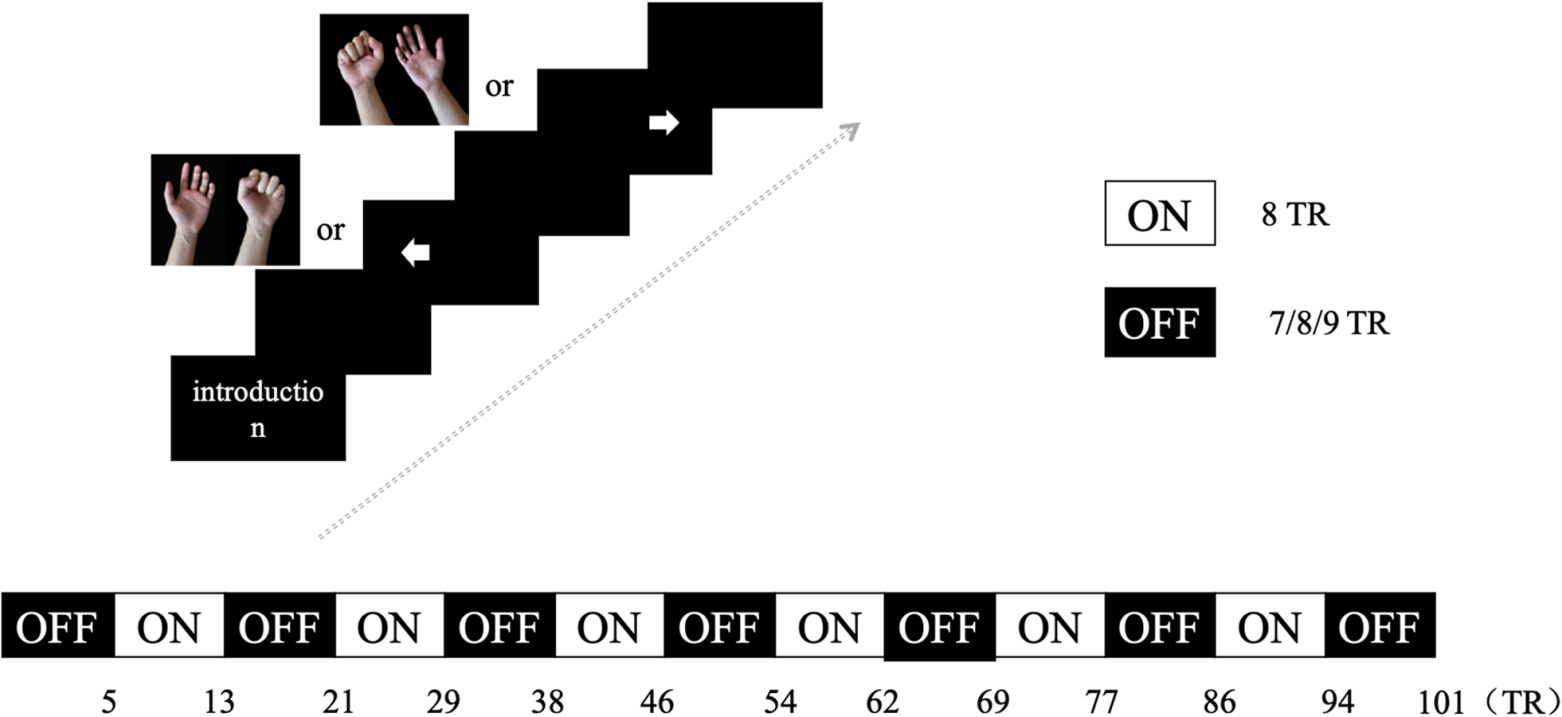
Schematic of the fMRI block design paradigm. All subjects performed two sessions of four tasks (**a** motor execution with left hands, **b** motor execution with right hands; **c** motor imagery with left hands, **d** motor imagery with right hands). During fMRI scanning, participants were asked to perform two different block design paradigms. For the first session (motor execution), the subjects grasped and relaxed the corresponding hands according to the prompts on the screen at a frequency of 1 Hz. For the second session (motor imagery), the subjects imagined the corresponding hand movements according to the arrows. Each “ON” block lasted for 20 s (8 TR) with the left and right hand displayed pseudorandomly, with intervals with an “OFF” block lasting 7–9 scans of blank screen displayed pseudorandomly. A total of 101 volumes were acquired per session

Prior to the commencement of the fMRI scans, individuals in the preparatory session engaged in fist-making motions and hand relaxation exercises. The resting-state functional MRI scan lasted for an estimated duration of 10 min. To reduce the likelihood of unexpected motion, participants were instructed to close their eyes and remain motionless. They all demonstrated compliance throughout the fMRI procedure of the resting-state. In the task-state fMRI scan, participants recline on their backs in a relaxed position, with cushion support near their hips. They were instructed to maintain a slightly bent elbow position based on individual comfort preferences and carry out hand movements or imagery as indicated by pictures shown. The sessions involved varying visual cues, with participants alternating between executing actual grasping motions or relaxation in response to images displaying closed fists or a relaxed hand, and mentally simulating a gripping action when presented with an arrow image. Individuals were instructed to complete two different block design tasks. During the first session (motor execution), participants executed hand movements in response to visual cues displayed on the screen at a frequency of 1 Hz. During the second session (MI), the participants mentally simulated the matching hand motions following the indicated arrows [9, 20]. Each block endured for 20 s. In each session, one hundred volumes were acquired while the left- or right-hand occurrences were generated in a pseudorandom manner. To avoid subjects forming expectations of the task, a blank screen was displayed at random intervals of 7–9 scans during the scanning procedure. Throughout the imaging process, the attending research team conducted observational checks to ensure that all individuals carried out the required tasks appropriately (Fig. 1).

### Data preprocessing and analysis

The spatial preprocessing and analysis procedures utilized the Statistical Parametric Mapping 12 (SPM12) software (Wellcome Trust Centre for Neuroimaging, London; https://www.fil.ion.ucl.ac.uk/spm/software/spm12/) carried out on the MATLAB 2014a platform developed by MathWorks in Natick, MA, USA. To standardize lesion locations across patients and establish consistent normalization criteria, we flipped the brain images of individuals with right-sided lesions over the mid-sagittal plane, ensuring that the affected hemisphere aligned with the left side of the brain for all subjects.

The analysis of resting-state data was performed with RESTplus V1.2 (the Resting-State fMRI Data Analysis Toolkit plus V1.2, http://restfmri.net/forum/RESTplusV1.2), to perform amplitude of low-frequency fluctuations (ALFF) or regional homogeneity (ReHo) computations. In line with our previous research [21, 22], we followed the same data processing protocol by discarding the initial ten volumes of each subject to reach signal equilibrium, then the processing procedure including: (1) time correction of slice scans; (2) the correction of head movement (all head movements were below 2.5 mm or 2.5° in any direction); (3) aligning the functional brain images to the standard EPI template through spatial normalization; (4) regression of nuisance variables, including the white matter and cerebral spinal fluid blood oxygen level-dependent (BOLD) signals and head motion with six motion profiles; (5) spatial smoothing using a Gaussian kernel with a full width at half maximum of 6 mm before the ALFF calculation and after ReHo calculation; (6) removal of linear trends; (7) ALFF and ReHo calculations for the traditional low-frequency band (0.01–0.08 Hz), and (8) transformation of both ALFF and ReHo values into a Z score (zALFF and zReHo)for further comparison between groups.

The manipulation of the task-state data before statistical analysis involved correcting time slices, motion correction, spatial normalization into a standard template in MNI space (using the T1 SPM template and resulting in voxels of 3 × 3 × 3 mm^3^), and adjusting for motion-related signal changes using the six realignment parameters in the design matrix. We applied a temporal high-pass filter with a 128 s cutoff and an AR(1) model for temporal autocorrelation [23] and conducted a 6 mm full width at half-maximum isotropic Gaussian kernel to smooth the normalized images.In our analysis of fMRI data, we utilized a mass-univariate approach based on general linear models (GLMs). We modeled task conditions of motor execution (ME), and motor imagery (MI) for both the unaffected hand (UH) and affected hand (AH) with the hemodynamic response function. Estimates for the contrast of interest were generated by incorporating session-type regressors and six motion parameters in the first-level models for individual participant images (for this contrast, we set the threshold significance at p < 0.001, uncorrected). We created group maps by applying a one sample t test.

### Statistical analyses

We evaluated differences between and within groups or contrast images at the second level for the test variables of fMRI data with two-sample *t* tests or paired samples *t* tests (with a significance level of p < 0.01, uncorrected, cluster > 10 voxels). We then overlaid the results on the standard Ch2 template. We recorded the brain regions with statistically significant differences and mapped the significant clusters to the AAL partition template. Visualization was achieved by MRIcroGL (https://www.nitrc.org/plugins/mwiki/index.php/mricrogl:MainPage).

We classified different brain areas between groups as areas of interest with REST software based on the ALFF/ReHo data. We obtained the mean value for each area of interest by calculating the average ALFF/ReHo value of all voxels. We assessed the correlations between the mean zALFF/zReHo values in multiple brain areas between groups and the associated behavioral performances by analyzing Pearson correlation coefficients with GraphPad Prism 8 (Graph Pad Software Inc., San Diego, CA, USA) (at a significance level of P < 0.05).

## Results

A total of 40 patients with poststroke upper extremity hemiparalysis were identified and included in this study. A previous study reported demographic and clinical characteristics, and identified a significant difference in the recovery of upper limb motor function [17].

### Group differences in brain activation in the block-design scan

**Fig. 2:**
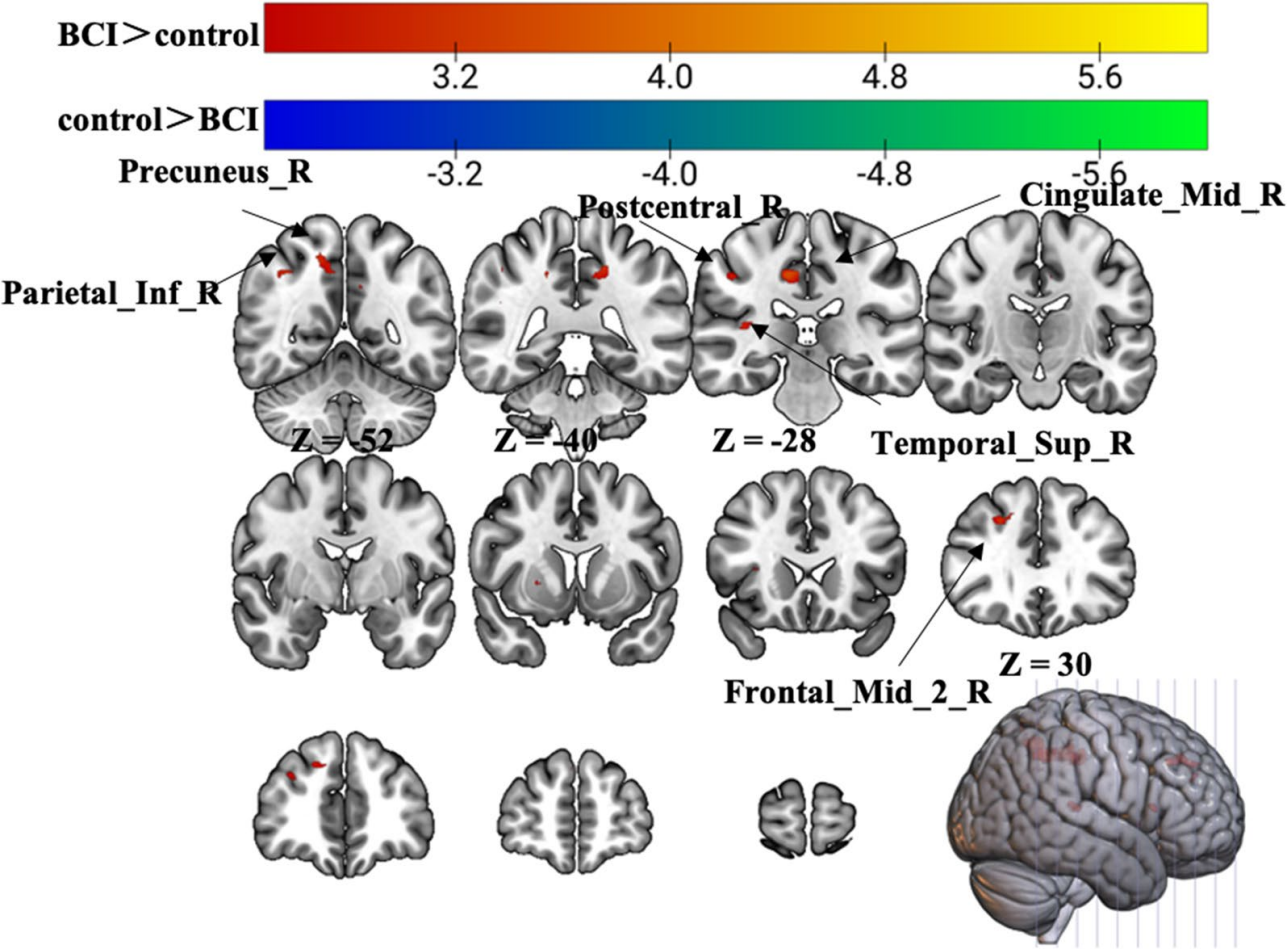
Group differences in brain activation area on task-state fMRI during motor execution with the affected hand. Warm tones represent greater brain activation in the BCI group than in the control group, while cold tones represent less brain activation in the BCI group than in the control group. The z value was the z-axis coordinate along the anterior–posterior axis referenced to a stereotaxic brain SPM152 template

**Fig. 3:**
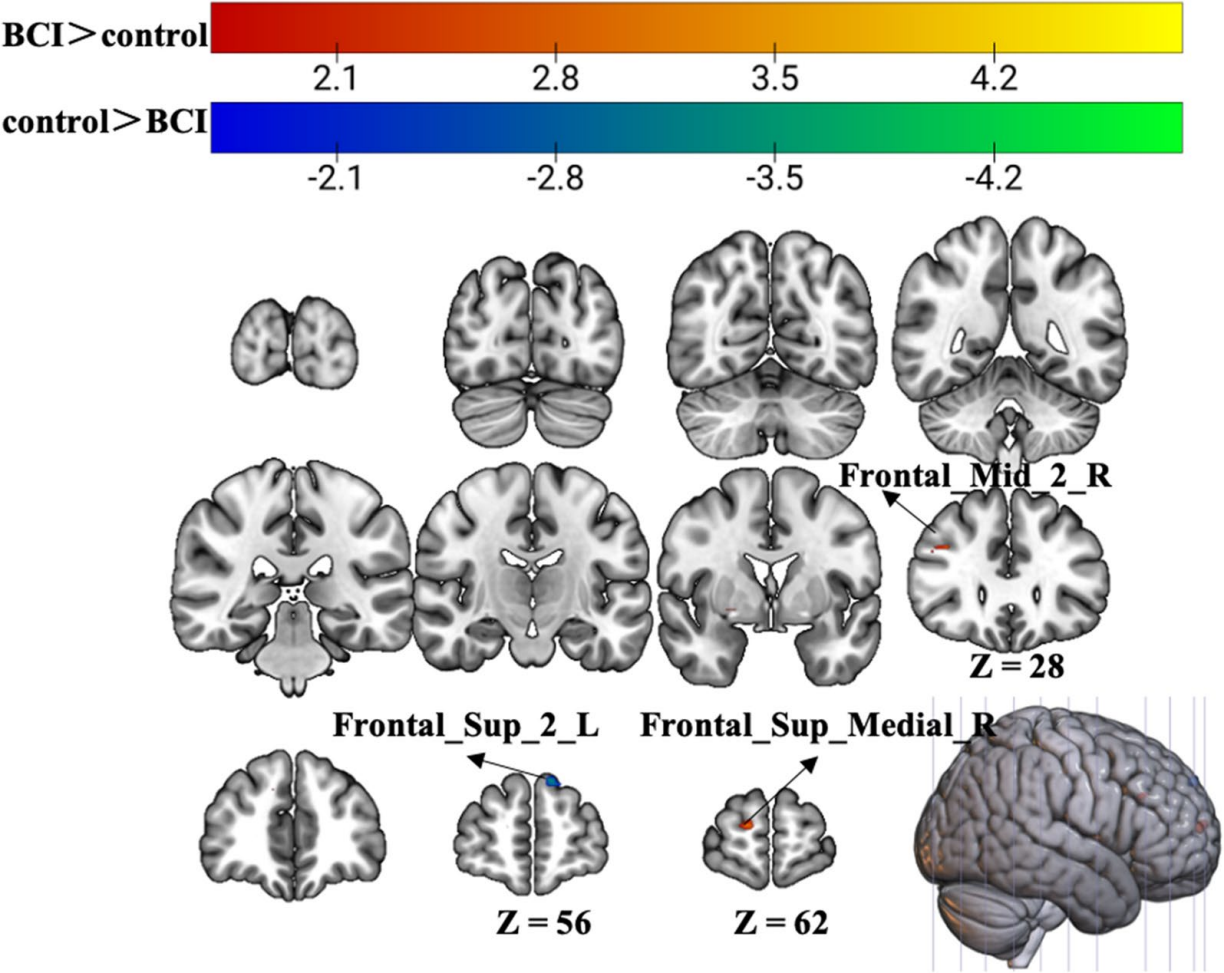
Group differences in brain activation area in task-state fMRI of motor imagery with the affected hand. Warm tones represent greater brain activation in the BCI group than in the control group, while cold tones represent less brain activation in the BCI group than in the control group. The z value was the z-axis coordinate along the anterior–posterior axis referenced to a stereotaxic brain SPM152 template

All the subjects suppressed unexpected movement and were compliant during the fMRI task. The group activation maps and the corresponding MNI coordinates of the activated brain regions during the different task conditions performed with the AH were determined. Figures 2 and 3 show the differences in whole-brain activation between the BCI group and the control group. Tables 1 and 4 summarize the corresponding MNI coordinates of the active brain regions during the different tasks performed with the AH.

**Table 1:** Group differences of brain activation area in task-state fMRI of motor execution

Brain regions	Extent	Cluster centroid (MNI)			t-value
		x	y	z	
Control group > BCI group					
None	–	–	–	–	–
BCI group > control group					
Cingulate_Mid_R	140	9	− 27	42	3.581
Precuneus_R	140	15	− 51	51	3.207
Parietal_Inf_R	54	36	− 48	39	3.432
Postcentral_R	54	45	− 30	39	3.121
Frontal_Mid_2_R	41	24	27	36	3.193
Temporal_Sup_R	18	39	− 30	12	3.125
Cingulate_Mid_L	10	− 6	− 15	45	2.893

*Cingulate_Mid_R* Middle Cingulate Gyrus, Right, *Precuneus_R* Precuneus, Right; *Parietal_Inf_R* Inferior Parietal Gyrus, Right, *Postcentral_R* Postcentral gyrus, Right; *Frontal_Mid_2_R* Middle Frontal Gyrus, Right, *Temporal_Sup_R* Superior Temporal Gyrus, Right, *Cingulate_Mid_L* Middle Cingulate Gyrus, Left

### Motor execution

There were significant changes in fMRI task activation for each group postintervention. Greater ME activation with the AH was observed in the BCI group than in the control group in the ipsilateral middle cingulate gyrus, precuneus, inferior parietal gyrus, postcentral gyrus, middle frontal gyrus, superior temporal gyrus, and contralateral middle cingulate gyrus (p < 0.01, uncorrected, cluster > 10 voxels) (Fig. 2, Table 1).

### Motor imagery

However, when imagining the grasping task with the affected hand, the BCI group exhibited greater activation in the ipsilateral superior frontal gyrus (medial) and middle frontal gyrus after treatment. However, activation of the contralateral superior frontal gyrus decreased in the BCI group relative to the control group (p < 0.01, uncorrected, cluster > 10 voxels) (Fig. 3, Table 2).

**Table 2:** Group differences of brain activation area in task-state fMRI of motor imagery

Brain regions	Extent	Cluster centroid (MNI)			t-value
		x	y	z	
*Control group > BCI group*					
Frontal_Sup_2_L	11	− 9	57	39	3.7056
*BCI group > control group*					
Frontal_Sup_Medial_R	20	15	60	12	3.822
Frontal_Mid_2_R	12	36	27	30	3.5447

*Frontal_Sup_2_L* Superior Frontal Gyrus, Left, *Frontal_Sup_Medial_R* Superior Frontal Gyrus (medial), Right, *Frontal_Mid_2_R* Middle Frontal Gyrus, Right

### Group differences in zALFF in the resting state

**Fig. 4:**
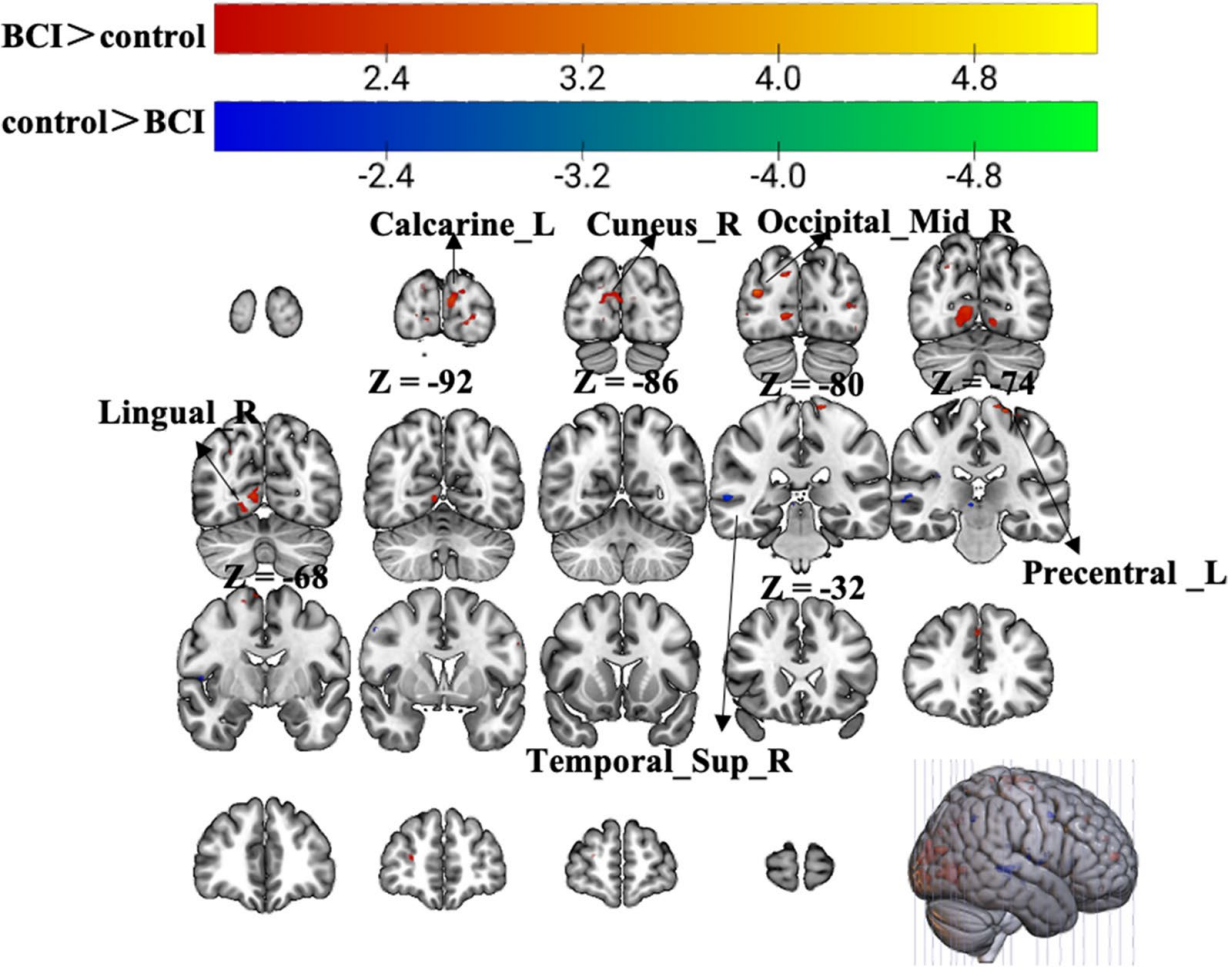
Group differences in zALFF in the resting-state scan. Warm tones represent greater zALFF values in the BCI group than in the control group, while cold tones represent lower zALFF values in the BCI group than in the control group. The z value was the z-axis coordinate along the anterior–posterior axis referenced to a stereotaxic brain SPM152 template

Statistical analysis revealed increased zALFF in multiple cerebral regions, including the contralateral precentral gyrus and calcarine, reflecting the enhanced activity of local brain regions and the ipsilateral middle occipital gyrus and cuneus (p < 0.01, uncorrected, cluster > 10 voxels). Additionally, we found decreased zALFF values in the ipsilateral superior temporal gyrus, which suggested that the activity of the ipsilateral superior temporal gyrus was weaker than that in the control group (Fig. 4, Table 3).

**Table 3:** Group differences of ALFF in resting-state scan

Brain regions	Extent	Cluster centroid (MNI)			t-value
		x	y	z	
Control group > BCI group					
Temporal_Sup_R	25	54	− 33	3	2.774
BCI group > Control group					
Precentral_L	41	− 21	− 27	69	4.225
Occipital_Mid_R	23	36	− 81	18	3.408
Calcarine_L	89	− 6	− 90	12	3.041
Cuneus_R	89	15	− 87	24	2.521
Occipital_Mid_R	28	− 18	− 90	− 6	2.974

*Temporal_Sup_R* Superior Temporal Gyrus, Right, *Precentral_L* Precentral gyrus, Left, *Occipital_Mid_R* Middle Occipital gyrus, Right, *Calcarine_L* Calcarine, Left, *Cuneus_R* Cuneus, Right

### Group differences in ReHo in the resting state

**Fig. 5:**
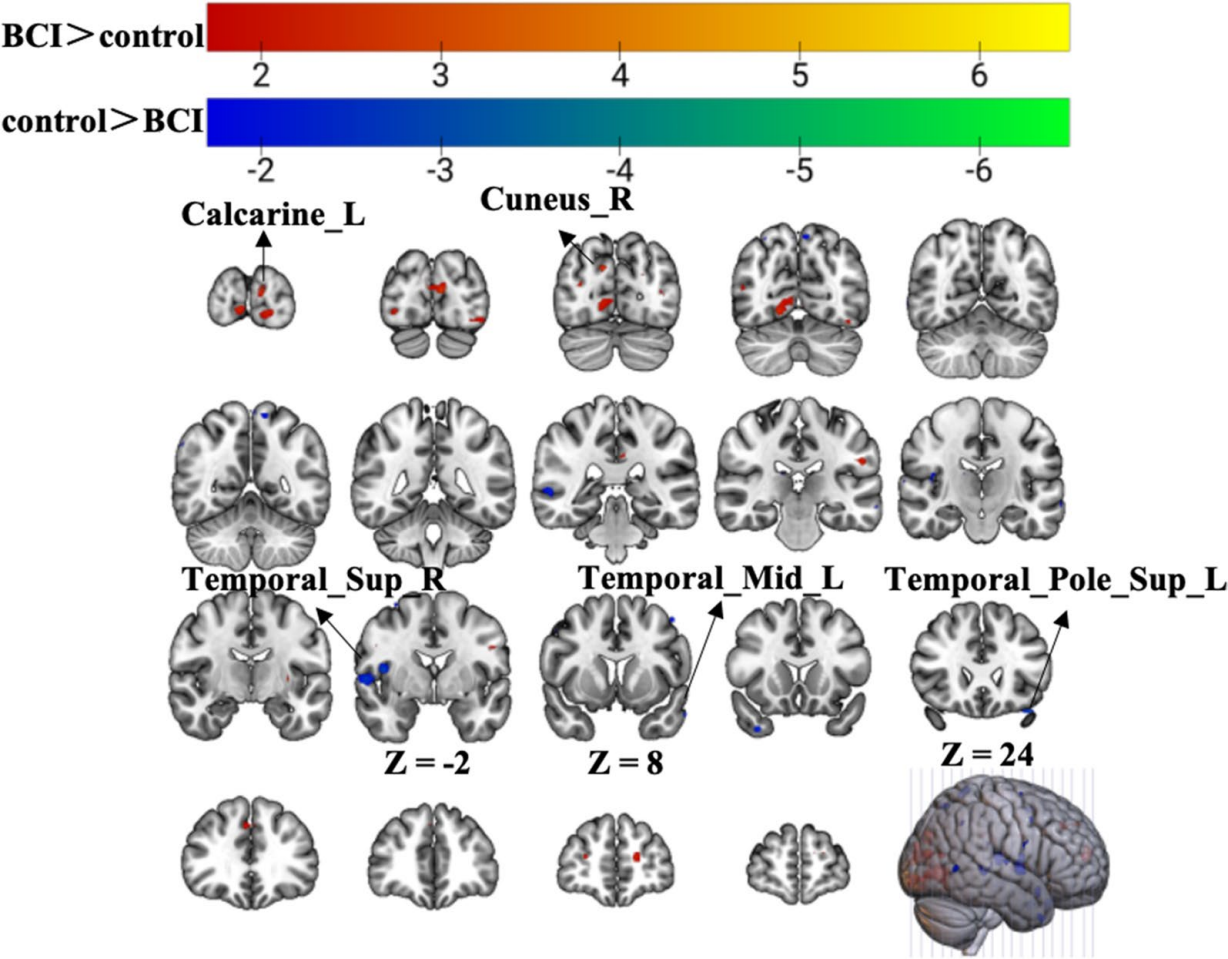
Group differences in zReHo in the resting-state scan. Warm tones represent greater zReHo values in the BCI group than in the control group, while cold tones represent lower zReHo values in the BCI group than in the control group. The z value was the z-axis coordinate along the anterior–posterior axis referenced to a stereotaxic brain SPM152 template

Compared with the control group, the BCI group had significantly greater zReHo values in the ipsilateral cuneus and contralateral calcarine, as shown in Fig. 5 (red) and Table 2. Moreover, the zReHo values of the contralateral middle temporal gyrus, temporal pole, and superior temporal gyrus in the BCI group were obviously lower than those in the control group, as shown in Fig. 5 (blue) and Table 4 (p < 0.01, uncorrected, cluster > 10 voxels).

**Table 4:** Group differences of ReHo in resting-state scan

Brain regions	Extent	Cluster centroid (MNI)			t-value
		x	y	z	
*Control group > BCI group*					
Temporal_Mid_L	10	− 57	6	− 27	− 3.957
Temporal_Pole_Sup_L	13	− 36	21	− 27	− 3.836
Temporal_Sup_R	10	57	− 3	3	− 2.994
*BCI group > control group*					
Cuneus_R	13	9	− 81	33	3.615
Calcarine_L	17	− 9	− 93	12	3.243

*Temporal_Mid_L* Middle Temporal Gyrus, Left, *Temporal_Pole_Sup_L* Temporal Pole (superior), Left, *Temporal_Sup_R* Superior Temporal Gyrus, Right, *Cuneus_R* Cuneus, Right, *Calcarine_L* Calcarine, Left

### Correlation analysis

**Fig. 6:**
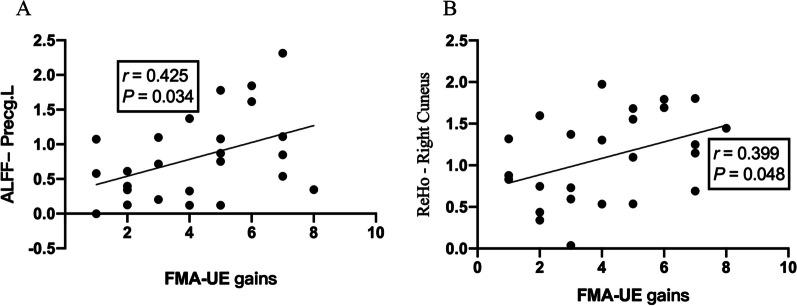
Correlation analysis between zALFF/zReHo and FMA-UE gains. The zALFF values of the ipsilesional primary motor cortex showed a trend toward a positive correlation with increased FMA-UE score (**A**). The zReHo values of the contralesional cuneus showed a trend toward a positive correlation with increased FMA-UE score (**B**)

In the group correlation analysis, the increase in clinical scale (FMA-UE) score was positively correlated with the mean zALFF of the contralateral precentral gyrus (r = 0.425, P < 0.05) (Fig. 6A) and the mean zReHo of the right cuneus (r = 0.399, P < 0.05) (Fig. 6B).

## Discussion

This study aimed to explore the effects of motor imagery (MI)-based brain-computer interface (BCI) rehabilitation programs on upper extremity and hand function in patients with chronic hemiplegia. We adopted a randomized controlled trial design and divided 46 eligible stroke patients into a BCI group and a control group. The Fugl-Meyer motor assessment (FMA) score was used as the primary outcome to evaluate the motor function of the upper extremity. We performed fMRI scans on patients before and after treatment, including in the resting state and task state. The task state was divided into four tasks: left-hand grasping, right-hand grasping, imagining left-hand grasping, and imagining right-hand grasping. Then, we used correlation analysis of clinical assessments and functional measures to assess meaningful differences. The BCI group had significant improvement in upper extremity motor function compared with the control group and exhibited different degrees of activation or inhibition in multiple brain regions. In addition, the clinical improvement in the BCI group was positively correlated with the zALFF of the contralateral precentral gyrus and the zReHo of the ipsilateral cuneus. These results suggest that BCI therapy is an effective and safe method for upper extremity rehabilitation after stroke, and its neural mechanism may be related to visual and spatial processing.

### Task-related fMRI

The dynamic engagement of multiple cognitive processes, including image preparation and/or execution, engagement with internal models of the body, semantic processing of auditory cues, assignment of attention to cued body locations or body properties, and corresponding actual sensations of upcoming stimuli and/or sensory memories, is involved in MI-BCI training. In this study, when performing the motor grasping task with the affected hand, the postcentral gyrus, middle frontal gyrus, middle cingulate gyrus, precuneus, inferior parietal gyrus, and superior temporal gyrus of the unaffected side of the BCI group exhibited better activation than did those of the control group after treatment.

There was significant activation of the postcentral gyrus and the middle cingulate gyrus on the affected side. The postcentral gyrus contains the primary somatosensory cortex, the main sensory receptive area for the sense of touch. The middle frontal gyrus is mainly composed of Brodmann Area 6 (BA6) and Brodmann Area 9 (BA9). BA6 is located directly in front of Brodmann Area 4 (BA4, primary motor cortex), which includes the premotor cortex and the supplementary motor area, and plays an important role in planning complex, coordinated movements. The BA9 area is composed of the dorsolateral and medial prefrontal cortex, which are involved in short-term memory, inferring spatial imagery, inferring intention and other cognitive activities. Therefore, after MI-BCI intervention, motor-related brain areas in the contralateral brain, mainly the postcentral gyrus and the middle frontal gyrus, are activated, thereby improving motor function.

Current fMRI research has brought attention to a particular cerebral network that is active during anticipatory thinking. Known as the default mode network (DMN), this complex network is composed of the medial prefrontal cortex (mPFC), the posterior cingulate cortex (PCC)/precuneus, the inferior parietal lobe, the lateral temporal cortex, and the hippocampal formation, which were essential brain regions involved in memory and cognitive processing. The DMN is typically segmented into anterior and posterior subsystems [24–26]. The anterior DMN includes the mPFC, the dorsal medial prefrontal cortex, the anterior cingulate cortex, the PCC, the anterior temporal lobe, the inferior frontal gyrus, and the lateral parietal cortex. In contrast, the posterior DMN is made up of the posterior cingulate cortex, the precuneus, the posterior inferior parietal lobule, and the angular, hippocampal, and temporal lobes [24–26]. The mPFC and the PCC serve as hub regions for these subsystems.

The DMN is usually inactive during tasks that demand focus on external stimuli [27, 28] but becomes active in states of rest or when engaging in internal mental activities like recalling personal history, understanding others' perspectives, introspective thinking, and envisioning the future [29, 30]. Thoughts about the future engage a variety of mental activities, including introspective cognition [31], a personal perception of time, and the construction of scenes (namely, the process of retrieving and combining elements from past experiences into a unified occurrence) [32, 33], all of which rely on a broad network of brain regions within the DMN. Imagining personal future scenarios tends to produce more pronounced responses in the ventral medial prefrontal cortex and posterior cingulate cortex than nonpersonal scenarios [34]. The precuneus has been recognized for its broader involvement in functions such as navigating space, behavior influenced by spatial cues, focusing on spatial details, learning visual words, sustaining working memory, executing visuospatial strategies, generating words, and identifying targets and new stimuli. It is involved in both sensory-motor processing and more complex cognitive and emotional tasks, including visualization, recalling episodic memories, and self-reflection. In particular, the dorsal posterior areas of the medial and lateral precuneus play a role in visual and motor imagery [35, 36].

The exact neural correlates of MI are unknown, but evidence suggests that both imagined and actual movements may involve the same internal mental representations or internal models of the body [37, 38]. Support for this theory of shared representations comes mainly from the parallel domain of MI [38]. The imagined and actual movements show clear similarities at the behavioral (e.g., similar duration), physiological (e.g., changes in heart rate), and neural levels (e.g., possibly activating the same neural substrates) [39–41]. These studies have been interpreted as evidence that imagined movements are simulations of internal models that track the state of our bodies during exercise [42]. Human neuroimaging studies have shown that during imagined and actual movement, overlapping brain regions, including the primary motor cortex, premotor cortex, supplementary motor cortex, primary sensory cortex, and other broad cortical regions, are activated [37, 43]. This finding points not only to a shared neural substrate for representing imagined and actual movements but also to similar internal models that may be used. Internal models may be involved in anticipated or planned activities (and/or in relation to the imagination).

Our study identifies potential areas involved in the effects of MI-BCI by quantitatively analyzing the activation of neural response clusters during actual ME and MI processes. The movement-related effects appear to be mediated by a range of other cognitive processes, leading to the activation of frontoparietal and visual area clusters.

### Resting-state fMRI

We examined changes in the amplitude and local coherence of low-frequency oscillations in patients undergoing MI-BCI therapy and conventional rehabilitation in the resting state. The results showed that BCI training significantly increased ALFF and ReHo values relative to those of patients who underwent conventional rehabilitation. ALFF is a functional index reflecting the strength of spontaneous neuronal activity, and ReHo reflects the consistency of neuronal activity. The positive correlation between the primary motor cortex of the affected side and improvements in motor function scores further confirmed that MI-BCI can better promote improvements in motor function in stroke patients. This finding is consistent with previous research results. Bajaj et al. [20] reported that the PMC and M1 play crucial roles in MI and ME tasks. Zhu et al. [44] studied visual feedback therapy (VFT) based on the mirror neuron theory. Compared with those in the conventional rehabilitation group, the motor function of the patients in the VFT group significantly improved after 8 weeks of recovery training, and fMRI revealed that the bilateral activation of the precentral gyrus, parietal lobe and auxiliary motor area was significantly greater in the VFT group than in the conventional rehabilitation group. The authors concluded that VFT is an effective method for improving upper extremity motor function and daily activities in stroke patients, promoting sensorimotor plasticity and behavioral changes in motor and sensory domains. This therapeutic mechanism promotes motor relearning by activating the mirror neuron system and motor cortex. Similarly, in a controlled study, Michielsen et al. [45] found that patients receiving mirror therapy had a shift in the balance of activation in the primary motor cortex from the unaffected side to the affected side, resulting in greater improvements in motor function.

Many BCI systems are controlled through MI, but these systems may not have been developed specifically for dyskinesia, which is independent of normal motor control [46]. Subsequent studies have demonstrated that patients can also achieve the same or similar control of BCI as healthy subjects, and by using MI techniques to reconstruct similar normal work environments and conditions, targeted training can improve patient outcomes [47].The degree of physical movement recovery may differ due to varying severity between subjects. Thus, although these early systems established a precedent for MI, more detailed research is needed to develop a standard protocol for training with exercise-oriented BCI devices [48]. Currently, BCI devices that rely on MI continue to be used for rehabilitation, and newer systems have also used BCI to incorporate actual movement into their protocols for better rehabilitation [49–51]. Researchers prefer the use of the BCI system to perform actual attempted movements in open-loop and closed-loop conditions rather than purely imagined movements, because the feedback provided by the device is fully controlled by neural signals and detected by EEG, with the computer generating real-time interfaces between stimuli. During training, patients perform closed-loop tasks during which real-time visual feedback is presented to help patients learn to modulate cortical activity during attempted movements of each hand [52].

In our study, consistent activity of the unaffected cuneiform lobe was positively associated with motor gain in patients. The cuneiform lobe is thought to be a traditionally functional brain region involved in visual processing, with high activity in the dorsal visual processing stream. The cortex around the calcarine fissure is the area where the primary visual cortex is concentrated. Likewise, the cuneiform lobe and lingual gyrus are both occipital lobes associated with visual stimuli and information processing. The lingual gyrus is an important structure of the occipital visual cortex that is involved in the regulation of visual stimuli and is related to complex image coding, visual memory processing, working memory, selective attention, word recognition, semantic processing, and visual information recognition of faces. The study revealed that the lingual gyrus was significantly activated during memory tasks, and the corresponding regions of the occipital lobe, including the lingual gyrus, exhibited task-selective memory effects when subjects were processing visual imagery tasks. The enhancement of the lingual gyrus signal may be related to the retrieval of relevant memories during the task, indicating that the lingual gyrus may be related to the hippocampus; it may also be related to the amygdala, because the activation of the lingual gyrus was confirmed when subjects performed a high-emotion word task. Our study thus confirmed that MI-BCI promotes visual stimulation and semantic integration-related changes in brain regions to improve motor function in patients.

## Conclusion

This parallel-group study demonstrated that MI-BCI therapy significantly improved motor function in chronic moderate-to-severe stroke patients. fMRI results suggested that the motor-related areas of the affected brain hemisphere and the regions that process visuospatial sensory tasks were significantly activated and were positively correlated with improvements in motor function. This recovery is associated with quantitative signatures of functional neuroplasticity. Importantly, the benefits of BCI intervention include the remodeling of brain regions related to motor and visuospatial information processing, which may have important implications for optimizing BCI protocols. The identification of these activated areas allow for the combination of BCI with conventional therapy and the individualization of interventions, enabling the closed-loop decoding of brain activity to play a key role in recovery.

## Limitation

Our study had several limitations in its design and execution that require attention. Firstly, the control group in the experimental phase did not receive an equal amount of treatment compared to the experimental group, which goes against randomized controlled trial standards. This discrepancy might have affected the reliability of our findings. Moreover, the short two-week treatment period and limited number of training sessions could have prevented significant changes in the participants, obscuring important findings. The constraints on participants’ hospital stays further restricted the possibility of extending the sessions or undertaking follow-up studies to uncover meaningful effects. The hand rehabilitation device we used was specifically designed for wrist and finger dorsiflexion, and its application in other muscle groups for motor rehabilitation of different body parts, such as elbows and shoulders, has not yet been explored. The primary outcome measure, FMA-UE, focuses more on upper limb motor function, and is less sensitive to hand function, necessitating further research. We used a significance level of p < 0.01 and a minimum cluster size of 10 voxels, but we did not perform multiple comparison correction for this level, which may have resulted in some overlapping or redundant findings. Additionally, the small participant sample, broad spectrum of post-stroke intervals, diverse stroke types, and lesion locations pose challenges to the comprehensive evaluation of treatment efficacy. Further research is needed to validate and refine the methodological framework of future studies in this domain.

## Data Availability

The datasets generated during and/or analyzed during the current study are available from the corresponding author upon reasonable request.

## Availability of data and materials

The raw data supporting the conclusions of this article will be made available by the authors, without undue reservation.

## References

[1] Shih JJ, Krusienski DJ, Wolpaw JR (2012). Brain–computer interfaces in medicine. Mayo Clin Proc.

[2] Nierhaus T, Vidaurre C, Sannelli C, Mueller K-R, Villringer A (2019). Immediate brain plasticity after one hour of brain–computer interface (BCI). J Physiol.

[3] He B, Baxter B, Edelman BJ, Cline CC, Ye W (2015). Noninvasive brain–computer interfaces based on sensorimotor rhythms. Proc IEEE Inst Electr Electron Eng..

[4] Angerhöfer C, Colucci A, Vermehren M, Hömberg V, Soekadar SR (2021). Post-stroke rehabilitation of severe upper limb paresis in Germany—toward long-term treatment with brain–computer interfaces. Front Neurol.

[5] Jiang Y, Yin J, Zhao B, Zhang Y, Peng T, Zhuang W, et al. Motor imagery brain–computer interface in rehabilitation of upper limb motor dysfunction after stroke. J Vis Exp. 2023. 10.3791/65405 37677045

[6] Liao W, Li J, Zhang X, Li C (2023). Motor imagery brain–computer interface rehabilitation system enhances upper limb performance and improves brain activity in stroke patients: a clinical study. Front Hum Neurosci.

[7] Sebastián-Romagosa M, Cho W, Ortner R, Sieghartsleitner S, Von Oertzen TJ, Kamada K (2023). Brain–computer interface treatment for gait rehabilitation in stroke patients. Front Neurosci.

[8] Grimm F, Naros G, Gharabaghi A (2016). Closed-loop task difficulty adaptation during virtual reality reach-to-grasp training assisted with an exoskeleton for stroke rehabilitation. Front Neurosci.

[9] Gerardin E, Sirigu A, Lehéricy S, Poline J-B, Gaymard B, Marsault C (2000). Partially overlapping neural networks for real and imagined hand movements. Cereb Cortex.

[10] Ang KK, Guan C, Chua KSG, Ang BT, Kuah CWK, Wang C (2011). A large clinical study on the ability of stroke patients to use an EEG-based motor imagery brain-computer interface. Clin EEG Neurosci.

[11] Ramos-Murguialday A, Broetz D, Rea M, Läer L, Yilmaz O, Brasil FL (2013). Brain–machine interface in chronic stroke rehabilitation: a controlled study. Ann Neurol.

[12] Miao Y, Chen S, Zhang X, Jin J, Xu R, Daly I (2020). BCI-based rehabilitation on the stroke in sequela stage. Neural Plast.

[13] Biasiucci A, Leeb R, Iturrate I, Perdikis S, Al-Khodairy A, Corbet T (2018). Brain-actuated functional electrical stimulation elicits lasting arm motor recovery after stroke. Nat Commun.

[14] Li M, Liu Y, Wu Y, Liu S, Jia J, Zhang L (2014). Neurophysiological substrates of stroke patients with motor imagery-based brain–computer interface training. Int J Neurosci.

[15] Mihara M, Hattori N, Hatakenaka M, Yagura H, Kawano T, Hino T (2013). Near-infrared spectroscopy-mediated neurofeedback enhances efficacy of motor imagery-based training in poststroke victims: a pilot study. Stroke.

[16] Kim T, Kim S, Lee B (2016). Effects of action observational training plus brain-computer interface-based functional electrical stimulation on paretic arm motor recovery in patient with stroke: a randomized controlled trial. Occup Ther Int.

[17] Ma Z-Z, Wu J-J, Hua X-Y, Zheng M-X, Xing X-X, Ma J (2023). Evidence of neuroplasticity with brain–computer interface in a randomized trial for post-stroke rehabilitation: a graph-theoretic study of subnetwork analysis. Front Neurol.

[18] Fugl-Meyer AR, Jääskö L, Leyman I, Olsson S, Steglind S (1975). The post-stroke hemiplegic patient. 1. A method for evaluation of physical performance. Scand J Rehabil Med.

[19] Sullivan KJ, Tilson JK, Cen SY, Rose DK, Hershberg J, Correa A (2011). Fugl–Meyer assessment of sensorimotor function after stroke: standardized training procedure for clinical practice and clinical trials. Stroke.

[20] Bajaj S, Butler AJ, Drake D, Dhamala M (2015). Brain effective connectivity during motor-imagery and execution following stroke and rehabilitation. NeuroImage Clin..

[21] Pu L, Zou Y, Wang Y, Lei J-L, Zhao X-N, Zeng X (2023). The relationship between processing speed and remodeling spatial patterns of intrinsic brain activity in the elderly with different sleep duration. Front Neurosci.

[22] Ma J, Hua X-Y, Zheng M-X, Wu J-J, Huo B-B, Xing X-X (2021). Spatial patterns of intrinsic brain activity and functional connectivity in facial synkinesis patients. Br J Neurosurg.

[23] Johnstone T, Walsh K, Greischar L, Alexander A, Fox A, Davidson R (2006). Motion correction and the use of motion covariates in multiple-subject fMRI analysis. Hum Brain Mapp.

[24] Damoiseaux JS, Beckmann CF, Arigita EJS, Barkhof F, Scheltens P, Stam CJ (2008). Reduced resting-state brain activity in the “default network” in normal aging. Cereb Cortex.

[25] Lei X, Zhao Z, Chen H (2013). Extraversion is encoded by scale-free dynamics of default mode network. Neuroimage.

[26] Lei X, Wang Y, Yuan H, Mantini D (2014). Neuronal oscillations and functional interactions between resting state networks. Hum Brain Mapp.

[27] Raichle ME, MacLeod AM, Snyder AZ, Powers WJ, Gusnard DA, Shulman GL (2001). A default mode of brain function. Proc Natl Acad Sci USA.

[28] Spreng RN, DuPre E, Selarka D, Garcia J, Gojkovic S, Mildner J (2014). Goal-congruent default network activity facilitates cognitive control. J Neurosci.

[29] Buckner RL, Andrews-Hanna JR, Schacter DL (2008). The brain’s default network: anatomy, function, and relevance to disease. Ann N Y Acad Sci.

[30] Andrews-Hanna JR, Reidler JS, Sepulcre J, Poulin R, Buckner RL (2010). Functional-anatomic fractionation of the brain’s default network. Neuron.

[31] Buckner RL, Carroll DC (2007). Self-projection and the brain. Trends Cogn Sci.

[32] Hassabis D, Maguire EA (2007). Deconstructing episodic memory with construction. Trends Cogn Sci.

[33] Schacter DL, Addis DR, Hassabis D, Martin VC, Spreng RN, Szpunar KK (2012). The future of memory: remembering, imagining, and the brain. Neuron.

[34] D’Argembeau A, Stawarczyk D, Majerus S, Collette F, Van der Linden M, Feyers D (2010). The neural basis of personal goal processing when envisioning future events. J Cogn Neurosci.

[35] Zhang S, Li CR (2012). Functional connectivity mapping of the human precuneus by resting state fMRI. Neuroimage.

[36] Cavanna AE, Trimble MR (2006). The precuneus: a review of its functional anatomy and behavioural correlates. Brain.

[37] Schmidt TT, Blankenburg F (2019). The somatotopy of mental tactile imagery. Front Hum Neurosci.

[38] Kilteni K, Andersson BJ, Houborg C, Ehrsson HH (2018). Motor imagery involves predicting the sensory consequences of the imagined movement. Nat Commun.

[39] Lotze M, Halsband U (2006). Motor imagery. J Physiol Paris.

[40] Papaxanthis C, Pozzo T, Skoura X, Schieppati M (2002). Does order and timing in performance of imagined and actual movements affect the motor imagery process? The duration of walking and writing task. Behav Brain Res.

[41] Chivukula S, Zhang CY, Aflalo T, Jafari M, Pejsa K, Pouratian N, et al. Neural encoding of actual and imagined touch within human posterior parietal cortex. eLife. 2021;10. 10.7554/eLife.61646 PMC7924956 33647233

[42] Jeannerod M, Decety J (1995). Mental motor imagery: a window into the representational stages of action. Curr Opin Neurobiol.

[43] Lucas MV, Anderson LC, Bolling DZ, Pelphrey KA, Kaiser MD (2015). Dissociating the neural correlates of experiencing and imagining affective touch. Cereb Cortex.

[44] Zhu M-H, Zeng M, Shi M-F, Gu X-D, Shen F, Zheng Y-P (2020). Visual feedback therapy for restoration of upper limb function of stroke patients. Int J Nurs Sci..

[45] Michielsen ME, Selles RW, van der Geest JN, Eckhardt M, Yavuzer G, Stam HJ (2011). Motor recovery and cortical reorganization after mirror therapy in chronic stroke patients: a phase II randomized controlled trial. Neurorehabil Neural Repair.

[46] Leuthardt EC, Schalk G, Wolpaw JR, Ojemann JG, Moran DW (2004). A brain–computer interface using electrocorticographic signals in humans. J Neural Eng.

[47] Wolpaw JR, McFarland DJ (2004). Control of a two-dimensional movement signal by a noninvasive brain–computer interface in humans. Proc Natl Acad Sci USA.

[48] Felton EA, Wilson JA, Williams JC, Garell PC (2007). Electrocorticographically controlled brain-computer interfaces using motor and sensory imagery in patients with temporary subdural electrode implants. Report of four cases. J Neurosurg.

[49] Prasad G, Herman P, Coyle D, McDonough S, Crosbie J (2010). Applying a brain–computer interface to support motor imagery practice in people with stroke for upper limb recovery: a feasibility study. J Neuroeng Rehabil.

[50] Takahashi M, Takeda K, Otaka Y, Osu R, Hanakawa T, Gouko M (2012). Event related desynchronization-modulated functional electrical stimulation system for stroke rehabilitation: a feasibility study. J Neuroeng Rehabil.

[51] Mukaino M, Ono T, Shindo K, Fujiwara T, Ota T, Kimura A (2014). Efficacy of brain–computer interface-driven neuromuscular electrical stimulation for chronic paresis after stroke. J Rehabil Med.

[52] Young BM, Nigogosyan Z, Remsik A, Walton LM, Song J, Nair VA, et al. Changes in functional connectivity correlate with behavioral gains in stroke patients after therapy using a brain–computer interface device. Front Neuroeng. 2014;7. 10.3389/fneng.2014.00025 PMC4086321 25071547

